# Impact TMPRSS2–ERG Molecular Subtype on Prostate Cancer Recurrence

**DOI:** 10.3390/life11060588

**Published:** 2021-06-21

**Authors:** Anastasiya A. Kobelyatskaya, Elena A. Pudova, Anastasiya V. Snezhkina, Maria S. Fedorova, Vladislav S. Pavlov, Zulfiya G. Guvatova, Maria V. Savvateeva, Nataliya V. Melnikova, Alexey A. Dmitriev, Dmitry Y. Trofimov, Gennady T. Sukhikh, Kirill M. Nyushko, Boris Y. Alekseev, Sergey V. Razin, George S. Krasnov, Anna V. Kudryavtseva

**Affiliations:** 1 Engelhardt Institute of Molecular Biology, Russian Academy of Sciences, 119991 Moscow, Russia; pudova_elena@inbox.ru (E.A.P.); leftger@rambler.ru (A.V.S.); fedorowams@yandex.ru (M.S.F.); vladislav1pavlov@gmail.com (V.S.P.); guvatova.zulfiya@mail.ru (Z.G.G.); msavv@email.com (M.V.S.); mnv-4529264@yandex.ru (N.V.M.); alex_245@mail.ru (A.A.D.); gskrasnov@mail.ru (G.S.K.); 2 Institute of Gene Biology, Russian Academy of Sciences, 119334 Moscow, Russia; sergey.v.razin@usa.net; 3 Gynecology and Perinatology named after Academician V.I. Kulakov, National Medical Research Center for Obstetrics, Ministry of Health of the Russian Federation, 117997 Moscow, Russia; d.trofimov@dna-tech.ru (D.Y.T.); g_sukhikh@oparina4.ru (G.T.S.); 4 National Medical Research Radiological Center, Ministry of Health of the Russian Federation, 125284 Moscow, Russia; kirandja@yandex.ru (K.M.N.); burn-katya2011@yandex.ru (B.Y.A.)

**Keywords:** prostate cancer, TMPRSS2–ERG molecular subtype, tumor recurrence, gene expression, RNA–Seq

## Abstract

Currently, seven molecular subtypes of prostate cancer (PCa) are known, the most common of which being the subtype characterized by the presence of the TMPRSS2–ERG fusion transcript. While there is a considerable amount of work devoted to the influence of this transcript on the prognosis of the disease, data on its role in the progression and prognosis of PCa remain controversial. The present study is devoted to the analysis of the association between the TMPRSS2–ERG transcript and the biochemical recurrence of PCa. The study included two cohorts: the RNA–Seq sample of Russian patients with PCa (*n* = 72) and the TCGA–PRAD data (*n* = 203). The results of the analysis of the association between the TMPRSS2–ERG transcript and biochemical recurrence were contradictory. The differential expression analysis (biochemical recurrence cases versus biochemical recurrence-free) and the gene set enrichment analysis revealed a list of genes involved in major cellular pathways. The *GNL3*, *QSOX2*, *SSPO*, and *SYS1* genes were selected as predictors of the potential prognostic model (AUC = 1.000 for a cohort of Russian patients with PCa and AUC = 0.779 for a TCGA–PRAD cohort).

## 1. Introduction

Currently, seven major molecular subtypes of prostate cancer (PCa), identified by the Cancer Genome Atlas Research Network, TCGA–PRAD project [1], are known. Four of the seven subtypes are characterized by the presence of fusion transcripts between the *TMPRSS2* exons and the exons of genes encoding members of the erythroblast transformation-specific (ETS) family of transcription factors: *ERG*, *ETV1*, *ETV4*, and *FLI1* (the frequency of the subtypes is 46%, 8%, 4%, and 1%, respectively). Three other subtypes are characterized by the presence of point mutations in one of the following genes: *SPOP*, *FOXA1*, or *IDH1* (the frequency of the subtypes is 11%, 3%, and 1%, respectively) [1,2]. Thus, about half of all PCa cases have a TMPRSS2–ERG fusion transcript, which is formed due to an intrachromosomal rearrangement leading to the fusion of two genes: *TMPRSS2* and *ERG*. The *TMPRSS2* gene is characterized by a higher expression level compared with that of *ERG* gene expression in the prostate tissue [3]; however, their fusion leads to a manifold increase in *ERG* expression [4,5,6]. Furthermore, a number of studies have described TMPRSS2–ERG-mediated feed-forward regulation of wild-type *ERG*, inducing its overexpression [7,8]. *ERG* is a pro-oncogenic transcription factor involved in the regulation of embryonic development, cell proliferation, and differentiation. Multiple increases in the expression of the *ERG* entail serious transcriptomic reprogramming and altered cell signaling (for example, activation of WNT/TGF-beta and NOTCH pathways). It is believed that the fusion of *TMPRSS2* and *ERG* is an early driver event of PCa tumorigenesis. The TMPRSS2–ERG fusion transcript is found in prostatic intraepithelial neoplasia (PIN); the presence of this fusion transcript is associated with an unfavorable prognosis and more aggressive PCa [9,10,11].

Predicting PCa recurrence is a nontrivial task, despite the existing stratification schemes for risk groups based on traditional clinical parameters, such as the Gleason score, lymphatic dissemination, and preoperative prostate-specific antigen (PSA). The most widely used method to classify patients is that of D’Amico, which identifies three risk groups: low, intermediate, and high [12]. However, these risk groups do not accurately reflect the likelihood of recurrence in PCa. Biochemical recurrence (BCR) is established at postoperative PSA ≥ 0.2 ng/mL and, as a rule, occurs only in some high-risk patients but can also be observed in some intermediate-risk patients [10,13,14], which are cases with an unfavorable prognosis. Therefore, it is necessary to search for more precise prognostic markers. The TMPRSS2–ERG fusion transcript can be a potential marker of an unfavorable prognosis in PCa.

To date, a number of studies have been published that confirm the association of TMPRSS2–ERG with an unfavorable prognosis. For example, a study of several cohorts of PCa patients found less recurrence-free survival (RFS) for TMPRSS2–ERG-positive PCa cases [15]. At the same time, an important aspect is the quantitative level of expression of the TMPRSS2–ERG fusion transcript and the *ERG* gene, not just their presence [16]. On the other hand, several studies have shown that TMPRSS2–ERG is a precursor of a favorable prognosis. A recent study showed better survival in TMPRSS2–ERG-positive patients than in TMPRSS2–ERG-negative patients [17]. Several other studies on numerous cohorts of PCa patients also did not reveal a direct relation between TMPRSS2–ERG gene rearrangement and BCR [18,19]. Thus, the data on the association of the TMPRSS2–ERG fusion transcript with BCR are contradictory, and its role in the progression of PCa remains unclear.

In this work, we studied the association between the presence and expression level of the TMPRSS2–ERG fusion transcript and BCR using a cohort of 72 PCa samples obtained from Russian patients. We also searched for potential prognostic markers based on differential gene expression for the TMPRSS2–ERG molecular subtype using RNA–Seq data from the cohort of Russian patients and The Cancer Genome Atlas project (TCGA–PRAD).

## 2. Materials and Methods

### 2.1. Materials

The study used two cohorts: 72 tumor samples of PCa obtained from Russian patients and RNA–Seq data of the TCGA–PRAD project. Tumor samples of PCa with paired adjacent morphologically normal tissues were collected and characterized at the P.A. Herzen of the Ministry of Health of Russia (Table 1). The patients provided written informed consent to participate in this study. Samples were collected from patients not undergoing neoadjuvant therapy. Each sample contained a minimum of 70% of tumor cells.

For differential expression analysis, we used RNA–Seq data for locally advanced PCa cases from TCGA–PRAD [20] obtained from patients without neoadjuvant therapy and belonging to the Caucasian population. For these cases, the disease recurrence status and tumor molecular subtype were known (Table 1).

### 2.2. Methods

#### 2.2.1. Isolation of RNA and Reverse Transcription

Total RNA was isolated from fresh, frozen samples using the MagNA Pure Compact RNA Isolation Kit (Roche, Basel, Switzerland) according to the manufacturer’s protocol. The concentration of the isolated RNA was determined on a Qubit 2.0 fluorometer (Thermo Fisher Scientific, Waltham, MA, USA) using a Qubit RNA BR Assay Kit (Thermo Fisher Scientific). The quality of the isolated RNA was assessed on an Agilent 2100 Bioanalyzer (Agilent Technologies, Santa Clara, CA, USA) using an Agilent RNA 6000 Nano Kit (Agilent Technologies). The RNA integrity number (RIN) was no less than 8.0. Reverse transcription was performed using Mint reverse transcriptase (Evrogen, Moscow, Russia) according to the manufacturer’s protocol.

#### 2.2.2. Quantitative PCR (qPCR)

The TaqMan Gene Expression Assays Hs03063375_ft (Thermo Fisher Scientific) was used to assess the expression level of the TMPRSS2–ERG fusion transcript. The *RPN1* gene [21] was used as a control gene (primer and probe sequences for *RPN1* [22]). qPCR was performed with an Applied Biosystems 7500 Real-Time PCR System (Thermo Fisher Scientific). The following process was used for amplification: 95 °C for 15 min; 40 cycles at 95 °C for 15 s; 60 °C for 60 s. Each qPCR reaction was repeated three times. To assess the level of expression, the method of relative measurements (ΔCT) was used and calculations were performed using the ATG program (Analysis of Transcription of Genes) [23].

#### 2.2.3. RNA Sequencing

The mRNA libraries were prepared using a TruSeq^®^ Stranded mRNA LT kit (Illumina, San Diego, CA, USA) according to the manufacturer’s protocol. Sequencing was performed on the NextSeq500 System (read length—75 nt, single-end mode) using the NextSeq 500/550 High Output Kit v2.5 (Illumina).

#### 2.2.4. Data Analysis

FastqQC (v.0.11.9, Cambridge, UK) [24] and Trimmomatic (v.0.33, Jülich, Germany) [25] were used for the quality control and trimming of reads, respectively. The STAR splice-aware aligner (v.2.7.1, Cold Spring Harbor, NY, USA) [26] was used to map the reads to the reference genome (GRCh37.p13, GENCODE, Cambridge, UK) [27]. FeatureCounts (Subread package v.1.6.4, Parkville, Australia) [28] was used to calculate the read counts per gene. Differential expression analysis was carried out in the R environment (v.3.6.3, Vienna, Austria) [29] using the edgeR package (v.3.24.3, NSW, Australia) [30]. Gene set enrichment analysis (GSEA) was performed using the clusterProfiler package (v.3.14.3, Guangzhou, China) with the Gene Ontology (GO), Kyoto Encyclopedia of Genes and Genomes (KEGG), and REACTOME databases [31]. The specificity and sensitivity of candidate genes predicting unfavorable prognosis were estimated by the random forest (RF) method and receiver operating characteristic (ROC) analysis with the area under the ROC curve (AUC) calculation using the randomForest (v.4.6-14, Rahway, NJ, USA) [32] and pROC (v.1.17.0.1, Geneva, Switzerland) packages [33]. Both cohorts (Russian patients and TCGA) were used to train and test the random forest model. Each cohort was split multiple times into two groups (70% tumors—training set; 30% tumors—testing set). The impact of TMPRSS2–ERG fusion presence on RFS was accessed by Kaplan–Meier survival analysis using survminer R package (v. 0.4.8, Montpellier, France) [34].

#### 2.2.5. Statistics

The analysis of associations between the presence/level of expression of TMPRSS2–ERG and BCR was performed in the R environment (v.3.6.3) [29]. Pearson’s chi-squared test (*χ*^2^) and odds ratio quantity (OR) were used to assess the relationship between the TMPRSS2–ERG transcript presence and the presence of biochemical recurrence. To assess the association between the TMPRSS2–ERG expression level and biochemical recurrence, the Mann–Whitney U-test (MW test) and fold change (FC) quantity were used. Differences were considered statistically significant at a *p*-value ≤ 0.05.

The quasi-likelihood (QLF test) and the MW tests were used to assess the significance of changes in gene expression. Genes that passed a QLF *p*-value ≤ 0.05 were considered differentially expressed.

## 3. Results

### 3.1. Expression of the TMPRSS2–ERG Fusion Transcript in PCa Samples

Using qPCR, we assessed the expression of TMPRSS2–ERG transcript in 72 tumor samples of PCa and in paired adjacent normal tissues obtained from Russian patients. In all tested samples of normal prostate tissue, the expression of the TMPRSS2–ERG fusion transcript was not detected. The expression of TMPRSS2–ERG transcript was detected in 50% (36/72) of PCa tumor samples.

Furthermore, the whole cohort was divided into two groups based on postoperative PSA values: (1) BCR (PSA ≥ 0.2 ng/mL) consisting of 13 samples, and (2) the biochemical recurrence-free (BRF, favorable prognosis) group (PSA < 0.2 ng/mL) consisting of 52 samples (seven cases lacked postoperative PSA values data).

For these two groups of samples, factor analysis of the association of TMPRSS2–ERG fusion transcript presence with BCR was performed using the *χ*^2^ test and OR value calculation. The OR value predicting the direction of changes was 3.56 with *χ*^2^ *p*-value = 0.165.

**Figure 1:**
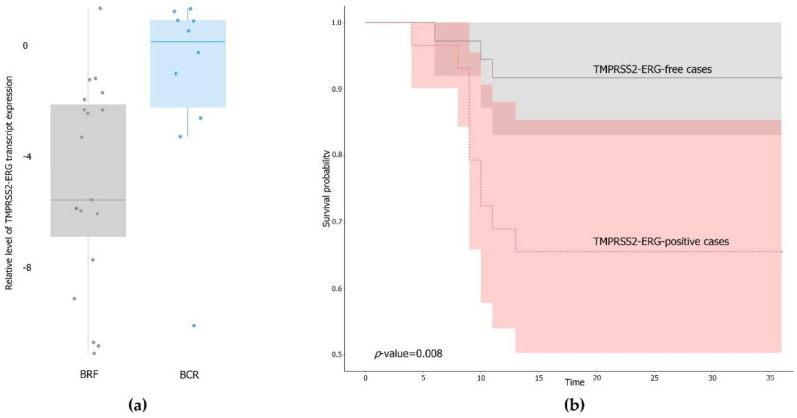
TMPRSS2–ERG fusion transcript in PCa samples. (**a**) Relative level of transcript expression in positive PCa samples. Y axis is relative level of TMPRSS2–ERG transcript expression (log2 transformation). BCR—biochemical recurrence group (blue), BRF—biochemical recurrence-free group (gray). (**b**) Recurrence-free survival (RFS) curves (with 95% CI—shaded areas) for TMPRSS2–ERG-free cases (grey) and TMPRSS2–ERG-positive cases (red). X axis is time in months, Y is the survival probability.

To evaluate the expression level of TMPRSS2–ERG transcript, ΔCT was calculated relative to the reference gene (*RPN1*) for TMPRSS2–ERG-positive samples (*n* = 36). By analogy with the whole cohort, these samples were also divided into two groups: (1) BCR (10 samples), and (2) BRF (19 samples). There was a significant increase in the expression level of the TMPRSS2–ERG fusion transcript in the BCR group by an average of 5.8 times (*p*-value = 0.04) (Figure 1a). Survival analysis showed significantly less RFS for TMPRSS2–ERG-positive cases (Figure 1b, *p*-value = 0.009).

Additionally, we analyzed the association between the presence of the TMPRSS2–ERG fusion transcript and BCR using a cohort of 203 PCa samples from the TCGA–PRAD project, which was also divided into the groups of BCR (*n* = 52) and BRF (*n* = 151). The OR value for this cohort was 0.83 with *χ*^2^ *p*-value = 0.80.

### 3.2. Differentially Expressed Genes and Significantly Enriched Pathways

Earlier, we performed an analysis of differentially expressed genes (DEGs) between groups of favorable and unfavorable prognoses for the lymph-node-negative LAPC and for the TMPRSS2–ERG-positive LAPC TCGA–PRAD cohort [35].

**Figure 2:**
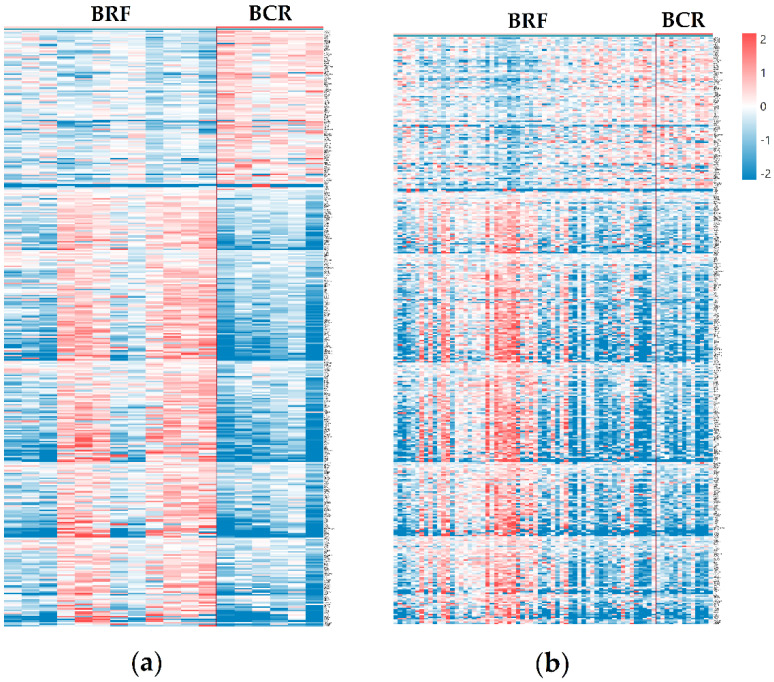
Heatmap of 388 differential expressed genes between BCR and BRF for TMPRSS2–ERG molecular subtype of the cohort of Russian patients and TCGA–PRAD cohort. (**a**) BCR versus BRF cases within TMPRSS2–ERG molecular subtype; (**b**) BCR versus BRF cases within TMPRSS2–ERG molecular subtype in TCGA–PRAD cohort. BCR—biochemical recurrence group, BRF—biochemical recurrence-free group. Red color indicates upregulated genes, blue—downregulated.

In the current study, we analyzed differential gene expression using an expanded cohort of PCa samples obtained from Russian patients and the TCGA–PRAD cohort. The following groups of comparisons were formed: (1) BCR versus BRF cases within the TMPRSS2–ERG molecular subtype and (2) BCR versus BRF cases within the TMPRSS2–ERG molecular subtype in the TCGA–PRAD cohort. As a result, 388 DEGs (QLF *p*-value ≤ 0.05, logCPM ≥ 3) were filtered for BCR within the TMPRSS2–ERG molecular subtype, which overlapped between the two studied cohorts (Figure 2, Supplementary Table S1). A total of 104 identified genes were characterized by an increase in expression in the BCR group within the TMPRSS2–ERG molecular subtype, while the expression levels of 284 genes decreased (Supplementary Table S1).

**Figure 3:**
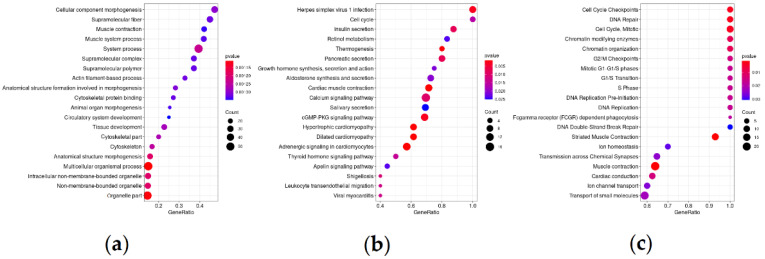
Dotplots showing top 20 enriched pathways. Y axes—pathways, X—gene ratio (number of input genes for this pathway/number of genes for this pathway), count—number of input genes for this pathway. (**a**) GO; (**b**) KEGG; (**c**) Reactome.

Using GSEA, enrichment with genes participating in several pathways related to the cytoskeleton, cell cycle, hormones, and secretion (insulin secretion, retinol, growth hormone pathways, calcium signaling), repair, and cell transport was revealed (Figure 3). The complete GSEA results are presented in Supplementary Table S2.

### 3.3. Four-Gene Prognostic Model

Among the 388 identified DEGs, seven candidate genes were highlighted as the most promising markers of unfavorable prognosis based on the distribution of differential expression between groups in both cohorts: *GNL3*, *ODF2*, *PAXBP1*, *QSOX2*, *SSPO*, *SYS1*, and *ZNF302* (Figure 2).

**Figure 4:**
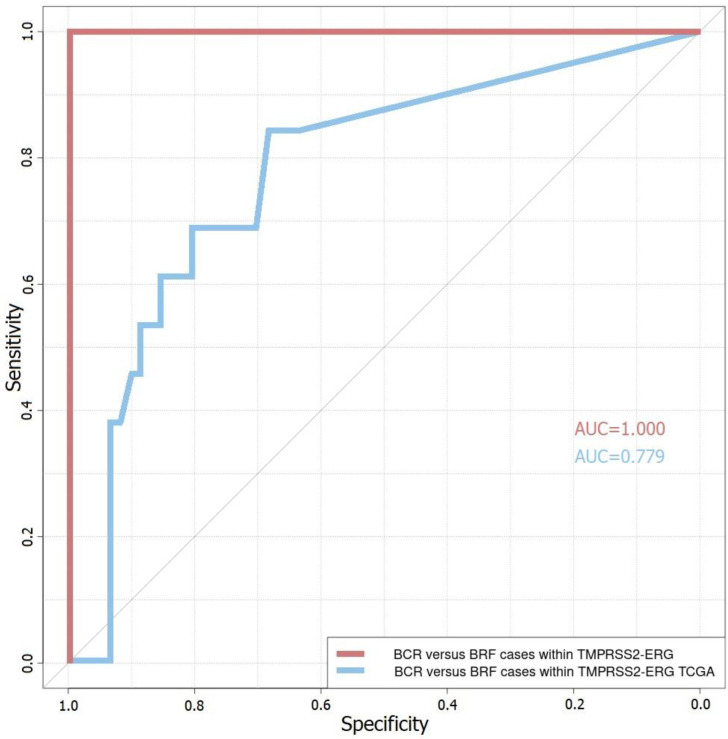
The ROC curves based on *GNL3*, *QSOX2*, *SSPO*, and *SYS1* gene expression within TMPRSS2–ERG-positive PCa. BCR—biochemical recurrence group, BRF—biochemical recurrence-free group; red line—ROC curve for Russian patient cohort (AUC = 1.000), blue line—TCGA–PRAD cohort (AUC = 0.779).

To determine a potential predictive model based on combinations of the aforementioned candidate genes, ROC analysis was performed. The combination of the genes *GNL3*, *QSOX2*, *SSPO*, and *SYS1* showed the best prognostic characteristics as a predictive model in both analyzed cohorts (AUC = 1 for the cohort of Russian patients, sensitivity = 1, specificity = 1; AUC = 0.779 for the TCGA cohort, sensitivity = 0.526, specificity = 0.870) (Figure 4). The results of other possible predictive models are presented in Supplementary Table S3. In our research, we identified *GNL3*, *QSOX2*, and *SSPO* as upregulated genes and SYS1 as downregulated for the BCR group within TMPRSS2–ERG-positive PCa (Table 2).

## 4. Discussion

Currently, the question of whether the presence and/or expression level of the TMPRSS2–ERG fusion transcript is a factor of unfavorable prognosis in PCa remains unclear. Several studies show contradictory results. In this work, we analyzed the association of the presence/expression level of the TMPRSS2–ERG fusion transcript with BCR in PCa.

A significant increase in the level of TMPRSS2–ERG expression was revealed in the BCR group in a cohort of Russian patients. At the same time, no statistically significant association between BCR and the presence of the TMPRSS2–ERG fusion transcript in the tumor was observed for both studied cohorts. However, less recurrence-free survival was observed for TMPRSS2–ERG-positive cases.

Since there is no unambiguous association of the TMPRSS2–ERG transcript with PCa recurrence, and the known markers of PCa do not have sufficient predictive power, the second part of our work was devoted to the search for potential prognostic markers of an unfavorable prognosis of PCa. Given the high biological heterogeneity of PCa [36], the search for potential markers was carried out within the TMPRSS2–ERG molecular sub-type, which is characterized by the highest occurrence (46% of all cases of PCa) [1].

Previously, we searched for potential prognostic markers for the cohort of Russian patients and the TCGA cohort [35,37]. However, we considered potential prognosis markers for a cohort of Russian patients without taking into account the TMPRSS2–ERG molecular subtype in view of the cohort size (*n* = 32). In addition, in the present study, a different study design was used for the TCGA cohort, including comparison groups and bioinformatic data processing.

As a result of the analysis, we obtained a list of DEGs (388 genes) common to both studied cohorts. The identified genes are involved in molecular pathways related to the regulation of the cytoskeleton, cell cycle and repair pathways, secretion pathways, and hormone signaling. The above pathways have also been associated with an unfavorable prognosis in lymph-node-negative PCa [35].

The following genes were selected as potential markers of unfavorable prognosis: *GNL3*, *ODF2*, *PAXBP1*, *QSOX2*, *SSPO*, *SYS1*, and *ZNF302*. Based on the results of the ROC analysis, the best primary predictive model that was identified relied on the combination of the *GNL3*, *QSOX2*, *SSPO*, and *SYS1* genes (AUC = 1 for the cohort of Russian patients; AUC = 0.779 for the TCGA cohort) for the TMPRSS2–ERG molecular subtype. At the same time, an increase in expression was noted for the *GNL3*, *QSOX2*, and *SSPO* genes, while the expression of the *SYS1* gene decreased in the group with an unfavorable prognosis.

The *GNL3* gene encodes for guanine nucleotide-binding protein-like 3, also known as the nucleostemin, which is required to maintain the proliferative capacity of stem cells. The nucleostemin is concentrated in the nucleus and extracellular matrix. It stabilizes the MDM2 protein by preventing its ubiquitination and, hence, proteasomal degradation. In this way, the *GNL3* protein may interact with p53 and may be involved in tumorigenesis. The *QSOX2* gene encodes quiescin sulfhydryl oxidase 2, a member of the sulfhydryl oxidase/quiescin-6 (Q6) family (QSOX1), which are involved in the sensitization of neuro-blastoma cells for IFN-gamma (IFNG)-induced cell death. The QSOX2 protein is localized as QSOX1 in the nucleus, Golgi apparatus, and extracellular matrix.

The *SSPO* (or *SSPOP*) is a pseudogene also known as SCO-spondin, which is involved in the modulation of neuronal aggregation. The *SSPO* may be involved in developmental events during the formation of the central nervous system. There are the metabolism of proteins and the O-glycosylation of TSR domain-containing proteins among its related pathways. 

The *SYS1* gene encodes the *SYS1* Golgi trafficking protein, which forms a complex with ADP-ribosylation factor-related protein ARFRP1 and targets ARFRP1 to the Golgi apparatus. The SYS1 protein is mostly localized in Golgi apparatus and cytosol.

A number of studies have also described the association of *GNL3* gene overexpression with tumor progression and poor survival in cancer of the prostate [38], stomach [39], colon [40], breast [41,42], and lung [43]. For three other identified genes (*QSOX2*, *SSPO*, and *SYS1*), we have described potential involvement in PCa for the first time.

In conclusion, the high expression level of the TMPRSS2–ERG fusion transcript was associated with unfavorable prognosis. Despite the lack of significant association of the unfavorable prognosis and presence of the TMPRSS2–ERG fusion transcript, comprehension of the TMPRSS2–ERG fusion transcript status in tumor is important to clarify which PCa prognostic model is appropriate for application. The most perspective prognostic model for TMPRSS2–ERG-positive PCa was based on expression profiles of *GNL3*, *QSOX2*, *SSPO*, and *SYS1* genes. The above model demonstrated sufficiently high predictive potential. However, further experimental research to validate this model on an expanded cohort by qPCR is needed.

## Figures and Tables

**Table 1:** Clinical and pathological characteristics of the studied cohorts.

Criterion		Cohort of Russian Patients with PCa	TCGA–PRAD Cohort
PCa samples, total Age (years), mean (range)		72	203
		63 (41–73)	62 (46–78)
pT, *n*	pT3a	35	98
	pT3b	37	105
pN, *n*	pN0	43	139
	pN1	29	64
pM, *n*	pM0	72	203
	pM1	0	0
Gleason score, *n*	6	7	8
	7	41	77
	8	10	30
	9	13	8
	10	1	2
Biochemical recurrence (PSA ≥ 0.2 ng/mL), *n*		13	63

**Table 2:** Differentially expressed genes associated with biochemical recurrence within TMPRSS2–ERG molecular subtype included in the prognostic model on ROC analysis. FC—fold change, logCPM—log2 counts per million, QLF—quasi-likelihood F-test, MW—Mann-Whitney U-test.

	Russian Patients				TCGA–PRAD			
Gene	FC	logCPM	QLF *p*-Value	MW *p*-Value	FC	logCPM	QLF *p*-Value	MW *p*-Value
*GNL3*	1.37	7.27	0.0095	0.0032	1.29	7.27	0.0043	0.0048
*QSOX2*	1.45	5.04	0.0086	0.0069	1.41	5.07	0.0005	0.0002
*SSPO*	2.65	4.62	0.0008	0.0083	2.08	3.32	0.0016	0.0012
*SYS1*	−1.33	4.90	0.0073	0.0001	−1.23	5.40	0.0068	0.0052

## Data Availability

PRJNA726203.

## Supplementary Materials

The following are available online at https://www.mdpi.com/article/10.3390/life11060588/s1, Table S1: DEA results, Table S2: GSEA results, Table S3: ROC results.
